# Effect of fortifying sorghum and wheat with Longhorn grasshopper (*Ruspolia differens*) powder on nutritional composition and consumer acceptability of biscuits

**DOI:** 10.1002/fsn3.4018

**Published:** 2024-02-13

**Authors:** Amos Kipkemoi Ronoh, Charlotte Atsango Serrem, Susan Balaba Tumwebaze, Gertrude Mercy Were

**Affiliations:** ^1^ Faculty of Agriculture Uganda Martyrs University Nkozi Uganda; ^2^ Institute of Food Bioresources Technology Dedan Kimathi University of Technology Nyeri Kenya; ^3^ Department of Consumer Sciences, School of Agriculture and Biotechnology University of Eldoret Eldoret Kenya; ^4^ Department of Forestry, Biodiversity & Tourism, School of Forestry, Environmental and Geographical Sciences Makerere University Kampala Uganda

**Keywords:** biscuits, children, fortification, *Ruspolia differens*, sorghum

## Abstract

This study aimed at improving the nutrient composition and protein quality of biscuits made from sorghum and wheat through fortification with Longhorn *Ruspolia differens* powder (RDP) for use as a supplementary food targeting children vulnerable to protein–energy malnutrition (PEM). Ten biscuit formulations were prepared by replacing a part of wheat and sorghum–wheat flours with 5, 15, 20, and 40% RDP. To establish the nutrient content of biscuits, proximate and mineral compositions were determined. The amino acid composition, reactive lysine and in vitro protein digestibility were determined for protein quality. Compositing wheat or wheat–sorghum biscuits with RDP increased the protein, fat, ash, and crude fiber by percentages as high as 118, 37, 133, and 573%, respectively. Mineral content increased with, iron, zinc, and potassium as high as 161, 219, and 169%, respectively. The lysine, reactive lysine and in vitro protein digestibility of the fortified biscuits increased significantly, relative to the 100% cereal biscuits. Fortification with RDP significantly improved the amino acid content of the biscuits but had a marginal effect on improvement of the lysine score and did not meet the reference pattern for children aged 3–10 years. The Protein Digestibility Corrected Amino Acid Score (PDCAAS) of wheat–sorghum and wheat biscuits improved by 6% to 47% and 2% to 33%, respectively, compared to the control biscuits. The fortified biscuits were liked by the consumers. The RDP‐fortified biscuits have the potential to alleviate PME in developing countries.

## Graphical abstract

Compositing wheat and sorghum–wheat biscuits with Longhorn grasshopper (*Ruspolia differens* powder (RPD)) improved the nutritional qualities of the biscuits. The fortified wheat and wheat–sorghum composite biscuits met half of the protein and the mineral requirements for children aged 0.5–10 years. The fortified wheat and wheat–sorghum composite biscuits were liked by consumers. The wheat and wheat–sorghum composite biscuits fortified with Longhorn grasshopper (*Ruspolia differens*) powder have considerable potential as protein‐rich supplementary foods to alleviate protein and energy malnutrition in children.

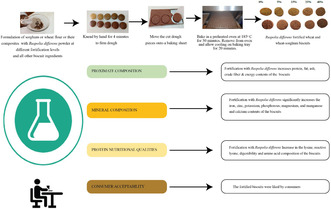

## 1 INTRODUCTION

Adequate protein intake to meet the requirement for optimal health, growth, and development of young children in developing countries continues to be a daunting nutritional challenge. This situation is exacerbated by unaffordability of high‐quality protein foods due to low income (Cornelsen et al., 2016), overpopulation, climate change (FAO/IFAD/UNICEF/WFP & WHO, 2021) and more recently, effects of the COVID‐19 pandemic (Carducci et al., 2021; Padhani et al., 2022). Consequently, recent global estimates show that in 2020, 149 million children under the age of 5 years were stunted while 45.5 million were wasted (FAO/IFAD/UNICEF/WFP & WHO, 2021). Of these, 41% and 27% stunted and wasted children, respectively, live in Africa (UNICEF/WHO & World Bank Group, 2021). Such children have increased susceptibility to impaired physical and cognitive development, morbidity, and mortality (Ahmed et al., 2020; Bosch‐Bayard et al., 2022).

In Sub‐Saharan Africa, dietary protein sources are primarily limited to cereal staples and to a less significant extent, animal sources (Schönfeldt & Gibson Hall, 2012). Sorghum (*Sorghum bicolor* (L.) Moench), the second most important cereal in Africa after maize, is consumed by more than 500 million people in over 30 countries (United Sorghum Checkoff Program & Lindsay, 2010). It is uniquely drought resistant and can grow where maize cannot, making it crucial for food security for vast populations in semiarid and arid areas. However, sorghum has poor protein quality because it is deficient in the essential amino acid lysine (Taylor & Duodu, 2017). Furthermore, the digestibility of sorghum seed storage protein kafirin is significantly reduced following wet cooking, during processing (Duodu et al., 2003), resulting in poor nutritional value. For instance, Maclean Jr. et al. (1981) established that feeding young children with sorghum porridge was associated with poor weight gain or loss. Consequently, efforts have been made in research to improve the protein quality of sorghum food products.

Food‐to‐food fortification of cereals with high‐quality proteins has been recommended as one of the sustainable ways of improving the protein quality and quantity to combat protein–energy malnutrition (PEM) (Chadare et al., 2019; FAO/WHO, 1994). Edible insects are among the alternative sources of high‐quality protein that could be used as additives and ingredients to fortify cereals due to their excellent amino acid profile and high digestibility, therefore enhancing nutritional qualities of plant‐based diets (Kim et al., 2022; Qian et al., 2022). Longhorn grasshopper (*Ruspolia differens*) is one of the edible insects commonly consumed in Uganda, Tanzania, and Kenya. It typically contains 43%–46% protein (Ssepuuya et al., 2019) and adequate ratios of all the eight indispensable amino acids (Fombong et al., 2017) comparable to those of pork loin muscle and broiler chicken meats (Ssepuuya et al., 2019). However, its use in the formulation of supplementary foods for alleviating PEM in school going children is limited. It can be used to fortify sorghum foods to improve their nutritional quality.

Biscuits are ready‐to‐eat foods prepared traditionally using wheat flour, fat, and sugar. However, technologies have been developed more recently to improve their properties in strategies to meet nutritional needs or prevent diet‐related diseases (Goubgou et al., 2021). Among children, biscuits are a popular snack, easy to carry (Manley, 2011), relatively cheap, have varied taste, are nutrient dense (Varghese & Srivastav, 2022), in addition to having a long shelf life (Manley, 2011). The wide consumption of biscuits makes them an ideal product for fortification (Kadam & Prabhasankar, 2010). Additionally, whole grain sorghum can be used to partially substitute highly processed wheat flour to improve the nutritional quality of biscuits.

Fortification of biscuits with edible insect powder has been investigated. For instance, Homann et al. (2017) who developed a biscuit for school going children fortified with 10% cricket powder reported improved protein quality, linoleic acid, vitamins A and B_12_, iron and zinc content, and acceptable taste. Specifically to sorghum, Awobusuyi et al. (2020) found that compared with 100% wheat flour, sorghum cookies fortified with termite powder had about double the quantity of protein and amino acids. However, fortification of sorghum‐based biscuits using *Ruspolia differens* powder (RDP) has not been studied. Therefore, the objective of this study was to formulate, develop, and determine the nutritional composition and consumer acceptability of biscuits made from sorghum and wheat, fortified with *Ruspolia differens* powder (RDP) for use as a protein‐rich supplementary food targeting children vulnerable to PEM.

## 2 MATERIALS AND METHODS

### 2.1 Materials

All‐purpose wheat flour “EXE” (Unga Limited, Eldoret, Kenya), pure white sugar “Nzoia Sugar” (Nzoia Sugar Company Ltd, Bungoma, Kenya), sunflower oil “SunGold” (Bidco Africa Ltd, Thika, Kenya), baking powder “Chapa Mandashi” (Kapa Oil Refineries Ltd, Nairobi, Kenya), and vanilla essence “Pradip” (Pradip Enterprises E.A. Ltd, Nairobi, Kenya) were all procured locally. Low tannin red sorghum was procured from Eldoret Municipal Market. Adult, live dewinged grasshoppers (*Ruspolia differens*) with appendages and antennae removed were procured from Busega Market on the outskirts of Kampala, Uganda during the November–December 2020 swarming season. They were packed in large plastic bags and transported to the School of Food Technology, Nutrition and Bio‐engineering, Makerere University, Kampala, Uganda within 15 min of procurement.

### 2.2 Processing of sorghum grain to flour

The sorghum was sorted to remove damaged grain, winnowed to separate chaff from grain, cleaned under running water on a sieve, and dried in the sun. The grain was then ground using a commercial hammer mill (Powerline®, BM‐35, Kirloskar, India) fitted with a 2.5 mm opening screen in Eldoret, Kenya. The flour was stored in airtight plastic containers at ambient temperature (25°C), until required.

### 2.3 Processing of fresh grasshopper (*Ruspolia differens)* to powder

Grasshoppers were put into large aluminum saucepans and thoroughly washed using clean running water, turning frequently until all soil and any remaining appendages and wings were properly dislodged. The clean grasshoppers were then drained of excess water in a large colander and chilled in large saucepans at −4°C for 1 h to kill the live insects before drying in a preheated air drier (B. Master, Tauro Comisano Vicentino (VI), Italy) at 65°C for 12 h. The dried insects were packaged in airtight polyethylene Ziploc bags and the temperature lowered by storing in a freezer at −18°C. Preliminary experiments showed that milling the insects at room temperature produced a paste. Therefore, the frozen grasshoppers were milled into a fine powder using an Electrical Powder Grinder Universal Mill (DE‐500g) with a fineness of 30 mm at a maximum speed of 2500 G.

The grasshopper powder was defatted using the method described by Zhao et al. (2016) with modification, using ethanol as an extraction solvent. The solvent‐to‐material ratio of 5 mL/g was used with 99.5% ethanol absolute. The insect powder–ethanol mixture was boiled in a water bath at 80°C for 30 min and allowed to settle for 5 min. The liquid was decanted and the filtrate retained. The residue was passed through a cheesecloth and wrung, until all liquid was exhausted. The powder that remained on the cheesecloth was put on a tray for 1 h to allow the ethanol to evaporate. The ethanol–oil phase was separated using a rotary evaporator (BM 100, Yamato Scientific Co. Ltd, Japan) with water bath set at 65°C, medium speed. The defatted powder was vacuum packed and kept at −18°C in polyethene Ziploc bags. These bags were then transported at ambient temperature (approximately 23°C) to University of Eldoret in Kenya, where they were stored in a freezer at −4°C until required.

### 2.4 Biscuit formulation

The concept for formulating the biscuit targeting school‐aged children was adopted from Serrem et al. (2011) to provide about half the protein requirement of 3–10 year‐old school going children. The Acceptable Macronutrient Distribution Range (AMDR) for protein–energy for prevention of PEM in this age group is 10 to 30 g protein per day (Institute of Medicine, 2005). This study therefore aimed at providing at least half, 13 g of protein per day, with 6.5 g provided in one biscuit. The basic formulation for the 100% wheat and 50:50 wheat–sorghum comprised 100 g flour, 25 g sugar, 30 g sunflower oil, and 6 g vanilla essence (Table 1). Water was dependent on the treatment and ranged from 17.8% (40% RPD: 60% wheat biscuits) to 27.1% (40% RPD: wheat–sorghum biscuits) of the total weight of ingredients.

**TABLE 1:** Formulation of the wheat, wheat–sorghum, *Ruspolia differens* powder (RDP), and composite biscuit doughs.

Ingredients	Fortification levels with *Ruspolia differens* powder									
	100% wheat					Wheat:sorghum (50:50)				
	0	5	15	25	40	0	5	15	25	40
*R. differens* powder (g)	0 (0)	5 (2.5)	15 (7.53)	25 (12.68)	40 (20.39)	0 (0)	5 (2.40)	15 (7.09)	25 (11.48)	40 (18.04)
Sorghum flour (g)	0 (0)	0 (0)	0 (0)	0 (0)	0 (0)	50 (24.55)	47.5 (22.76)	42.5 (20.08)	37.5 (17.23)	30 (13.53)
Wheat flour (g)	100 (49.73)	95 (47.45)	85 (42.67)	75 (38.03)	60 (30.58)	50 (24.55)	47.5 (22.76)	42.5 (20.08)	37.5 (17.23)	30 (13.53)
Sugar (g)	25 (12.43)	25 (12.49)	25 (12.55)	25 (12.68)	25 (12.74)	25 (12.27)	25 (11.98)	25 (11.81)	25 (11.48)	25 (11.27)
Sunflower oil (g)	30 (14.92)	30 (14.99)	30 (15.06)	30 (15.21)	30 (15.29)	30 (14.73)	30 (14.37)	30 (14.17)	30 (13.78)	30 (13.53)
Baking powder (g)	0.1 (0.05)	0.2 (0.1)	0.2 (0.1)	0.2 (0.1)	0.2 (0.1)	0.7 (0.34)	0.7 (0.34)	0.7 (0.33)	0.7 (0.32)	0.7 (0.32)
Vanilla essence (g)	6 (2.98)	6 (3)	6 (3.01)	6 (3.04)	6 (3.06)	6 (2.95)	6 (2.87)	6 (2.83)	6 (2.75)	6 (2.72)
Water (g)	40 (19.89)	39 (19.47)	38 (19.08)	36 (18.26)	35 (17.84)	42 (20.61)	47 (22.52)	50 (23.61)	56 (25.73)	60 (27.06)
Total dough wt (g)	201.1 (100)	200.2 (100)	199.2 (100)	197.2 (100)	196.2 (100)	203.7 (100)	208.7 (100)	211.7 (100)	217.7 (100)	221.7 (100)

*Note*: Figures in parentheses are percentages.

### 2.5 Preparation of biscuits

First, all the dry ingredients including flour, *Ruspolia differens* powder, sugar, and baking powder were sifted into a mixing bowl and mixed using a wooden spoon for 3 min. Then, oil, vanilla, and water were added gradually and the mixture kneaded for 4 min by hand to firm dough. The dough was divided into two portions, then manually sheeted on a steel tray to a height of 5 mm using a wooden rolling pin and cut into circular shapes using a 6.5‐cm diameter biscuit cutter. The excess dough was removed and added to the second portion of the dough to be rolled. The surface of each biscuit dough was pricked with a metal fork making holes to let moisture escape and avoid cracking of biscuits during baking. The dough pieces were then transferred onto a baking sheet and baked in a preheated air circulation oven at 185°C for 30 min. The baked aroma associated with baked goods and change in color to light brown were used as indicators that the biscuits were properly baked. After removal from the oven, the biscuits were cooled for 20 min at ambient temperature (23°C), packed in Ziploc bags, and stored in a freezer at −4°C to increase the shelf life until required.

### 2.6 Proximate analyses

The Association of Official Analytical Chemists (AOAC) International (1995) methods were used for proximate analyses. The oven drying procedure, Method 934.1, was used to determine the moisture content. Micro Kjeldahl, Method 992.23, was used to determine crude protein (N X 6.25). Soxhlet extraction, Method 920.29, was used to determine the crude fat content, while dry ashing, Method 923.03, was used to determine the ash content. Carbohydrate content was computed by difference, and energy computed using Atwater conversion factors (FAO, 2003). Dietary fiber (DF) was determined using the method described by Kirk and Sawyer (1991), while the crude fiber content was determined using AOAC International (1995) Method 922.06.

### 2.7 Mineral composition

Zinc (Zn), iron (Fe), magnesium (Mg), calcium (Ca), phosphorus (P), and manganese (Mn) were analyzed using atomic absorption spectrophotometry (AAS) (AOAC International, 1995) Method 985.35. The samples that had been digested for crude protein were atomized and their concentrations read off against the standards for each mineral. The concentration was obtained in milligrams per liter (mg/L).

### 2.8 Protein quality

The lysine content and that of other essential amino acids were determined after acidic and alkaline hydrolysis of the fat‐free sample and subsequent separation and quantification of the amino acids by a Waters Acquity Ultra‐Performance Liquid Chromatography (UPLC) H‐class with modifications according to AOAC Standard Method Performance Requirements (SMPR®) 2017.011 methods (AOAC, 2017). The amino acid score was determined as a ratio of milligrams per gram (mg/g) of the essential amino acid in the test protein to milligrams per gram (mg/g) of the same amino acid in the reference protein (WHO, 2007). The reference amino acid scoring pattern used was the amino acid requirement of preschool children aged 3–10 years (WHO, 2007). The reactive lysine content was determined by a rapid dye‐binding lysine method, as modified by Kim et al. (2007) using Crocein Orange G dye (70% dye content) (Fluka grade 27965: Sigma–Aldrich, Buchs, Switzerland). The pepsin digestion method of Hamaker et al. (1987) was used to determine the in vitro protein digestibility. Samples (200 mg) were digested with P7000‐100G pepsin, activity 863 units/mg protein (Sigma–Aldrich, St. Louis, MO, USA), for 2 h at 37°C. The residual protein was determined by the micro Kjeldahl method.

### 2.9 Consumer acceptability

Consumers (110), comprising 41% females and 59% males, aged 20–47 years and who were students or staff of the University of Eldoret were recruited through an advert on the notice boards. The panelists were required to be consumers of sorghum, biscuits, and animal proteins. Additionally, they were asked to fill a consent form informing them about the ingredients in the samples and to ascertain their commitment to participate in evaluation of the 10 biscuit samples. The evaluation session was carried out in the Foods Laboratory of the Department of Family and Consumer Sciences at the University of Eldoret. Each consumer was provided with all the 10 biscuit samples, each wrapped in clear plastic cling film, labeled with three‐digit random numbers, at ambient temperature (approximately 23°C). The samples were placed on a white tray accompanied with a bowl of carrots and a glass of distilled water to cleanse their palates before and in between tasting the samples. The consumers were asked to rate their degree of liking of appearance, aroma, flavor, color, and texture on a 9‐point hedonic scale, where 1 = dislike extremely, 5 = neither like nor dislike, and 9 = like extremely.

### 2.10 Ethical considerations

Ethical approval for this study was granted by the Mount Kenya University Institutional Ethics Review Committee (Application Approval Number: 1131). The National Commission for Science, Technology and Innovation (NACOSTI) issued a research license (number: NACOSTI/P/22/16130). Before evaluation, consumers submitted an informed, written consent.

### 2.11 Experimental design and statistical analyses

All the experiments were designed as single‐factor experiments in a completely randomized design. The levels of RDP substitution comprised the treatments and all tests were done in triplicate. The data were subjected to one‐way analysis of variance (ANOVA). Where the F‐ratio was significant, all pairwise differences between the treatment means were compared using Fisher's least significant difference (LSD) at *p* < .05. Statistical analyses were performed using Minitab Release 18 Software (Minitab Inc., Pennsylvania, USA).

## 3 RESULTS AND DISCUSSION

### 3.1 Proximate composition

Table 2 shows the proximate composition of the biscuits and flours. Wheat–sorghum biscuits had lower moisture contents (3%–5%) compared to wheat biscuits (3%–6%). The hydrophobic characteristic of sorghum kafirins compared to that of hydrophilic wheat proteins (Duodu et al., 2003) may explain this finding. As the baking temperature increased, prolamins in wheat absorbed water while kafirins in sorghum probably expelled water (Belton et al., 2006). The higher moisture content in wheat was probably due to damaged starch, high protein, and pentosans that absorb once, twice, and ten times their weights in water, respectively. The reduction in the wheat–sorghum biscuits can be explained by the hydrophobic characteristic of sorghum kafirins that probably expelled water during baking (Arepally et al., 2020). The significant reduction in moisture contents of both biscuits may also be attributed to reduction in the starch content and increase in the amount of insoluble dietary fiber, which could have influenced the water‐holding capacity of the biscuits (Sudha et al., 2007). Similar values have been reported in insect‐based biscuits. Akande et al. (2020) developed high‐energy biscuits from silk worm pupae and locust powders and found a moisture content of 3.6% to 4%. A similar finding was also reported by Serrem et al. (2011) who found that sorghum biscuits fortified with defatted soy flour had a significantly lower moisture content compared to wheat biscuits. According to the World Food Programme (2021) commodity specification for high‐energy biscuits for use in international food assistance programs, the moisture content should be 4.5% maximum. Results showed that all the fortified biscuits met this requirement. The low moisture content implies that the biscuits are shelf stable.

**TABLE 2:** The effect of fortifying sorghum and wheat with *Ruspolia differens* powder (RDP) on proximate composition of biscuits (g/100 g) dry weight basis.

Flour/biscuits	Moisture	Protein (N × 6.25)	Fat	Ash	Crude fiber	Carbohydrate^1^	Energy^2^ (kJ/g 100 g)	Dietary fiber
Flour								
Sorghum flour	9.48 ± 0.4^b^	11.22 ± 0.7^j^	3.40^i^ ± 0.3^i^	1.54 ± 0.3^bc^	2.25 ± 0.2^f^	72.11 ± 0.7^a^	1523^i^	6.23 ± 0.1^j^
Wheat flour	10.8 ± 0.7^a^	13.69 ± 0.3^h^	2.17^j^ ± 0.1^j^	0.51 ± 0.1^h^	0.85 ± 0.0^i^	71.99 ± 0.8^a^	1516^i^	11.53 ± 0.2^ef^
*Ruspolia differens* powder	7.42 ± 0.8^c^	49.69 ± 0.6^a^	21.7^f^ ± 0.2^f^	3.98 ± 0.4^a^	12.07 ± 0.2^a^	5.14 ± 0.9^j^	1734^h^	13.98 ± 0.4^c^
Wheat:RDP biscuits								
100:0	6.41 ± 0.4^d^	12.97 ± 0.2^i^	19.30 ± 0.9^h^	0.76 ± 0.1^g^	0.84 ± 0.0^i^	59.72 ± 0.8^b^	1943^g^	11.37 ± 0.3^f^
95:5	4.31 ± 0.1^ef^	14.90 ± 0.4^g^	20.97 ± 0.6^g^	0.81 ± 0.1^g^	1.38 ± 0.1^h^	57.64 ± 0.9^c^	2004^f^	12.32 ± 0.1^d^
85:15	4.08 ± 0.0^fg^	18.27 ± 0.3^e^	23.87 ± 0.5^d^	0.96 ± 0.1^fg^	2.58 ± 0.1^e^	50.25 ± 0.3^e^	2045^ab^	13.68 ± 0.3^c^
75:25	3.73 ± 0.0^gh^	25.13 ± 0.2^c^	24.66 ± 0.2^b^	1.16 ± 0.2^ef^	3.51 ± 0.3^d^	41.80 ± 0.5^g^	2049^ab^	14.57 ± 0.1^b^
60:40	3.23 ± 0.1^i^	28.01 ± 0.2^b^	26.38 ± 0.3^a^	1.28 ± 0.2^de^	5.65 ± 0.2^b^	35.47 ± 0.7^i^	2055^a^	15.70 ± 0.0^a^
Wheat–sorghum:RDP biscuits								
100:0	4.56 ± 0.0^e^	11.60 ± 0.5^j^	23.27 ± 1.4^e^	1.06^ef^ ± 0.1	1.64 ± 0.1^g^	57.87 ± 1.3^c^	2039^bc^	8.45 ± 0.3^i^
95:5	4.48 ± 0.0^e^	13.91 ± 0.3^h^	23.27 ± 0.1^e^	1.15^eg^ ± 0.1	2.21 ± 0.0^f^	54.97 ± 0.3^d^	2029^cd^	9.27 ± 0.2^h^
85:15	3.84 ± 0.0^gh^	17.44 ± 0.5^f^	23.87 ± 0.5^d^	1.23^e^ ± 0.1	3.51 ± 0.0^d^	50.41 ± 0.5^e^	2023^d^	10.67 ± 0.1^g^
75:25	3.54 ± 0.0^hi^	20.33 ± 0.7^d^	23.93 ± 0.1^cd^	1.46^cd^ ± 0.1	4.30 ± 0.12^c^	46.44 ± 0.8^f^	2019^de^	11.82 ± 0.2^e^
60:40	3.17 ± 0.1^i^	25.29 ± 0.7^c^	24.47 ± 0.1^bc^	1.77^b^ ± 0.2	5.60 ± 0.0^b^	39.70 ± 0.8^h^	2009^ef^	12.41 ± 0.0^d^

*Note*: Values are means ± standard deviation of three determinations. Values with the same letter superscript on the same column are not significantly different at (*p* < .05), as assessed by Fisher's least significant difference (LSD).

Abbreviation: RDP, *Ruspolia differens* powder.

^1^ Calculated as total carbohydrate by the difference method (FAO, 2003) where % carbohydrates = 100 − (% fat + % moisture + % ash (minerals) + % protein + % crude fiber) in 100 g of food.

^2^ Calculated by multiplying with Atwater's conversion factor (FAO, 2003) where energy (kJ) = (% carbohydrates × 16.736 kJ/g) + (% protein × 16.736 kJ/g) + (% oil × 37.656 kJ/g).

The fat content in wheat and wheat–sorghum biscuits increased significantly with increase in the RDP content in the composite flours. There was a 9% to 37% and 21% to 27% increase in the fat content of wheat and wheat–sorghum biscuits, respectively, compared to 100% wheat biscuits. This may be attributed to the high (22%) amount of fat in the RDP. This finding corroborates that of Awobusuyi et al. (2020) who found that fortification of sorghum biscuits with termite powder at 20% to 40% increased the fat content by 56% to 97%. Notably, *Ruspolia differens* is rich in essential fatty acids, such as oleic, palmitic, linoleic, linolenic, and arachidonic acids (Fombong et al., 2017), which are beneficial for growth and development of young children (Huffman et al., 2011). The fat content of 23%–24% in the fortified sorghum biscuits is within the FAO/WHO (1994) recommended range of 10–25 g oil per 100 g of foods meant for supplementary feeding of young children. The wheat biscuits had similar requirements, except for 40% RDP that surpassed and had a fat content of 26%.

Replacement of cereal flour with RDP ranging from 5% to 40% increased the ash content of wheat and wheat–sorghum biscuits by 7% to 68% and 39% to 133%, respectively, compared to the 100% cereal biscuits. The increase may be explained by the higher ash content of 4% of RDP compared to the two cereals with less than 2%. *Ruspolia differens* has high levels of iron, zinc, and phosphorus and moderate amounts of calcium, magnesium, and sodium (Ssepuuya et al., 2019). Similar results were reported by Haber et al. (2019) for increase in the ash content of wheat bread enriched with grasshopper powder by 26% to 37%.

The carbohydrate content decreased significantly as the levels of RDP increased in both biscuits. There was a 3.5% to 42% reduction in the carbohydrate content of wheat as the RDP levels increased compared to 100% wheat biscuits, whereas in the wheat–sorghum biscuits, there was 26% to 31% reduction in carbohydrate content compared to 100% wheat–sorghum biscuits. The reduction probably resulted from the lower (5%) carbohydrate content of the RDP. Since edible insects have been shown to have a low carbohydrate content (Nowak et al., 2016), compositing with RDP diluted the carbohydrate content of biscuits. Reduction in the carbohydrate content of baked goods as a result of enrichment with insect powder has been reported. Indriani et al. (2020) reported 14.2% to 44% reduction in the carbohydrate content of cakes fortified with 10% to 30% Bombay locust powder. Similarly, Awobusuyi et al. (2020) found a 57% to 68% reduction in carbohydrate content of sorghum biscuits fortified with termite meal.

The crude fiber content of wheat and wheat–sorghum biscuits increased significantly as the fortification levels with RDP increased. For instance, there was 64% to 573% increase in the crude fiber content of wheat biscuits as RDP levels increased from 5% to 40% compared to the control biscuits. This can be attributed to the high (12%) levels of crude fiber in the RDP. *Ruspolia differens* is a rich source of insoluble fiber from chitin exoskeleton (Ssepuuya et al., 2017). Furthermore, the process of defatting grasshopper powder as was done in this study leads to a significant increase in the fiber content, as demonstrated by Haber et al. (2019). Similar findings on increase in crude fiber content have been reported by Osimani et al. (2018) and Haber et al. (2019) while using cricket and grasshopper powders, respectively. The FAO/WHO (1994) Codex Committee recommends that foods for preschool children should contain 5 g of crude fiber maximum per 100 g of dry food. The crude fiber content of biscuits in this study ranged from 1.3% to 5.6% in wheat biscuits and 2.2% to 5.6% in wheat–sorghum biscuits. This shows that all of the biscuit formulations were within the recommended range, except the 40% RDP biscuits.

The dietary fiber significantly increased with increase in the RDP substitution. There was a 7% to 36% increase in dietary fiber content of wheat biscuits while in the sorghum biscuits, there was a 9.7% to 47% increase as a result of fortification. The high amount of dietary fiber in RDP (14%) is the possible cause of this finding. Generally, the wheat‐based biscuits had a higher amount of DF compared to the wheat–sorghum biscuits. This is because wheat flour had almost double the amount of DF compared to sorghum flour. According to Ibarra‐Herrera et al. (2020), the major dietary fiber in edible grasshoppers is insoluble dietary fiber that includes cellulose, hemicellulose, and lignin molecules. Similarly, Dewi et al. (2020) also found 77% to 217% increase in the dietary fiber content of biscuits fortified with 5% to 10% wood grasshopper powder.

The energy content increased with the level of RDP substitution in comparison to the flours. The energy density of the biscuits in this study was enhanced by the inclusion of sunflower oil in the formulation, in addition to fat sourced from RDP. This is because fat provides higher energy density of 37 kJ/g. The formulated biscuits contained about 2003 kJ to 2055 kJ and 2009 kJ to 2039 kJ for wheat and wheat–sorghum, respectively. This met the recommended minimum value of 1800 kJ/100 g for high‐energy biscuits used for school feeding programs as recommended by World Food Programme (2021). High‐energy density is essential for sparing protein for synthesis and repair of body tissues and also to provide energy for catch‐up growth among malnourished children (Michaelsen et al., 2009).

Compositing wheat and wheat–sorghum biscuits with RDP markedly increased the protein content of the biscuits. Replacement of cereal flour with 5, 15, 25, and 40% RDP increased protein content by 15, 41, 94, and 116%, respectively, in wheat‐based biscuits and 20, 50, 75, and 118%, respectively, in wheat–sorghum biscuits compared to the 100% cereal biscuits. This increase is probably due to the high protein content of RDP (50%). Several authors have reported similar results on the fortification of cereal‐based baked goods with edible insects. Niaba et al. (2013) found 9.6% to 21.7% increase in the protein content of sorghum‐based biscuits made from defatted termites. In another study, Biró et al. (2020) found 18% to 55% protein increase in oat biscuits fortified with 5% to 15% cricket powder.

#### 3.1.1 Percentage contribution of energy and protein content of sorghum and wheat biscuits fortified with RDP per 100 g toward RDA of children aged 0.5–10 years

Table 3 shows the contribution of the wheat–sorghum and wheat biscuits fortified with RDP per 100 g toward the Recommended Daily Allowance (RDA) of protein and energy for children aged 0.5–10 years. Results show that 100 g of the unfortified biscuits did not meet the protein RDA of the young children, particularly 8–10 years by half. However, the fortified biscuits meet the protein RDA needs of the children between 50%–180% and 53%–200% for the wheat–sorghum and wheat biscuits, respectively. For example, the 40% RDP wheat–sorghum and wheat biscuits met the protein RDA by 90 and 100%, respectively. This shows that 100 g of the biscuits was able to sufficiently meet the protein needs of the children. Furthermore, the percentage contribution toward the protein decreased as the age of the children increased to 10 years. The energy contribution also reduced with age from 57.3% to 54% at 0.5–1 year and from 24.4% to 24% at 7–10 years in the wheat–sorghum biscuit. A similar pattern was observed with the wheat‐based biscuits. The energy contribution increased from 54.6% to 57.8% at 0.5–1 year and from 23.2% to 24.6% at 7–10 years. The reduction in energy and protein can be attributed to increased requirements for metabolism, growth, and development (Michaelsen et al., 2009). Similar findings to this research work have been reported by Rapando et al. (2020) while working with fermented maize meal snacks fortified with soybean for school feeding programs. Furthermore, Dewi et al. (2020) found that consumption of one serving size of biscuits fortified with wood grasshopper flour contributed to the adequacy of protein per day by 24%–38% RDA of children aged 12–24 months.

**TABLE 3:** Percentage contribution of energy and protein content of sorghum and wheat biscuits fortified with *Ruspolia differens* powder (RDP) per 100 g toward RDA of children aged 0.5–10 years.

Nutrient	Age group (years)	RDA^1^	% contribution of wheat–sorghum and wheat biscuits fortified with RDP per 100 g toward RDA									
			Wheat–sorghum biscuits					Wheat biscuits				
			0	5	15	25	40	0	5	15	25	40
Energy (kJ/day)	0.5–1	3556.4 (850)	57.3	57.1	56.9	56.7	54.5	54.6	56.3	57.5	57.6	57.8
	2–3	5439.2 (1300)	37.4	37.3	37.1	37.1	36.9	35.7	36.8	37.6	37.7	37.8
	4–6	7531.2 (1800)	27.1	26.9	26.9	26.8	26.7	35.7	36.8	37.6	37.7	37.8
	7–10	8368 (2000)	24.4	24.2	24.2	24.1	24	23.2	23.9	24.4	24.5	24.6
Proteins (g/day)	0.5–1	14	82.9	99.3	124.6	145.2	180.6	92.6	106.4	130.5	179.5	200
	2–3	16	72.5	86.9	109	127.1	158.1	81.1	93.1	114.2	157.1	175.1
	4–6	24	48.3	57.9	72.7	84.7	105.3	54.0	62.1	76.1	104.7	116.7
	7–10	28	41.4	49.7	62.3	72.6	90.3	46.3	53.2	65.3	89.8	100.0

Abbreviation: RDA, recommended dietary allowance.

^1^ Food and Nutrition Board (1989). Figures in parentheses are energy/kcal/day.

Fortifying the sorghum and wheat biscuits at 40% RDP significantly increased the biscuits’ contribution of protein from 27% to 81% and 57% to 100%, respectively, above the RDA for children 0.5–3 years old. For children aged 4–10 years, fortification of sorghum and wheat biscuit with 40% RDP contributed 90%–105% and 100%–117%, respectively, toward protein RDA for children of age 4–10 years. The contribution of protein above the RDA for 0.5–3‐year‐olds is within tolerable limits and not toxic, as the recommended intake should not be more than double the RDA (Food and Nutrition Board, 1989). From these findings, the fortified biscuits more than adequately meet half of the protein requirements for children aged 0.5–10 years.

### 3.2 Mineral composition

The results of the mineral content of the biscuits and flours are shown in Table 4. The iron and zinc contents of the biscuits significantly increased with increase in the substitution levels of RDP. Compared to the 100% wheat biscuits, there was 11.5% to 161% increase in iron content of wheat biscuits, whereas in the wheat–sorghum biscuits, there was 18.2% to 73.4% increase as compared to the 100% wheat–sorghum biscuits. The zinc content increased in the wheat biscuits by 199% to 219% compared to 100% wheat biscuits, while in the wheat–sorghum biscuits, there was 32% to 160% increase compared to the 100% wheat sorghum biscuits. The significantly higher levels of iron and zinc in the sorghum biscuits compared to the wheat biscuits can be attributed to sorghum flour, which had almost double the amount of iron and zinc compared to wheat flour. The increase in the mineral content as a result of fortification may be ascribed to *Ruspolia differens* that contains high iron and zinc (Ssepuuya et al., 2019). These findings corroborate those of Awobusuyi et al. (2020) who also observed a significant increase in zinc and iron in sorghum‐based cookies fortified with termite powder.

**TABLE 4:** The effect of fortifying wheat–sorghum and wheat biscuits with defatted *Ruspolia differens* powder (RDP) on the mineral composition (mg/100 g).

Flour/biscuits	Iron	Zinc	Potassium	Phosphorus	Magnesium	Manganese	Calcium
Flour							
Sorghum flour	5.47 ± 0.2^g^	6.74 ± 0.2^d^	1.32 ± 0.6^bc^	0.48 ± 0.0^b^	205.92 ± 1.6^a^	1.61 ± 0.0^k^	67.63 ± 1.0^a^
Wheat flour	3.80 ± 0.2^h^	3.74 ± 0.1^i^	0.64 ± 0.1^c^	0.33 ± 0.0^d^	136.96 ± 0.8^b^	3.96 ± 0.1^c^	34.53 ± 0.9^j^
*RDP*	14.36 ± 0.3^a^	16.74 ± 0.3^a^	2.86 ± 0.9^a^	0.89 ± 0.0^a^	34.21 ± 0.7^i^	2.32 ± 0.0^j^	25.23 ± 0.2^k^
Wheat:RDP biscuits							
100:0	3.65 ± 0.3^h^	2.79 ± 0.3^j^	0.64 ± 0.3^c^	0.27 ± 0.1^f^	135.87 ± 2.9^h^	3.86 ± 0.0^d^	34.46 ± 0.0^j^
95:5	4.07 ± 0.0^h^	3.34 ± 0.2^i^	0.69 ± 0.23^c^	0.28 ± 0.0^def^	136.68 ± 0.2^h^	3.89 ± 0.0^d^	36.02 ± 0.5^i^
85:15	6.94 ± 0.1^f^	5.33 ± 0.1^g^	0.65 ± 0.1^c^	0.29 ± 0.0^def^	142.55 ± 0.6^g^	4.05 ± 0.0^c^	37.44 ± 0.2^h^
75:25	7.32 ± 0.4^f^	7.67 ± 0.0^d^	0.78 ± 0.1^c^	0.32 ± 0.0^d^	144.59 ± 1.6^g^	4.49 ± 0.1^b^	39.85 ± 0.2^g^
60:40	9.54 ± 0.4^d^	8.89 ± 0.2^c^	1.72 ± 1.5^b^	0.39 ± 0.0^c^	151.62 ± 1.1^f^	4.73 ± 0.1^a^	43.85 ± 1.7^f^
Wheat–sorghum:RDP biscuits							
100:0	8.09 ± 0.1^c^	4.42 ± 0.2^h^	0.88 ± 0.1^bc^	0.27 ± 0.0^ef^	169.71 ± 2.9^e^	2.69 ± 0.0^i^	50.27 ± 0.0^e^
95:5	9.54 ± 0.4^d^	5.83 ± 0.1^f^	0.99 ± 0.1^bc^	0.29 ± 0.0^def^	173.64 ± 0.6^d^	2.84 ± 0.0^h^	52.07 ± 0.2^d^
85:15	11.09 ± 0.2^c^	7.67 ± 0.3^d^	0.94 ± 0.4^bc^	0.31 ± 0.1^de^	176.04 ± 0.7^d^	3.08 ± 0.0^g^	55.77 ± 0.2^c^
75:25	12.80 ± 0.1^b^	8.58 ± 0.3^c^	1.17 ± 0.1^bc^	0.32 ± 0.0^d^	181.63 ± 1.6^c^	3.23 ± 0.0^f^	56.03 ± 0.9^c^
60:40	14.03 ± 0.6^a^	11.47 ± 0.3^b^	1.26 ± 0.2^bc^	0.39 ± 0.0^c^	186.31 ± 0.6^b^	3.56 ± 0.0^e^	61.48 ± 0.7^b^

*Note*: Values are means ± standard deviation of three determinations. Values with the same letter superscript on the same column are not significantly different at (*p* < .05), as assessed by Fisher's least significant difference (LSD).

Abbreviation: RDP, *Ruspolia differens* powder.

The potassium, phosphorus, magnesium, manganese, and calcium contents of the biscuits increased with RDP substitution. For instance, there was an 8% to 169% increase in the potassium content of wheat biscuits, and a 13% to 43% increase in the wheat–sorghum biscuits, relative to their control biscuits. The increase in the minerals was significantly different between the biscuit grain types, with sorghum–wheat biscuits having higher values compared to wheat biscuits. This may be explained by the higher mineral content in sorghum flour compared to wheat flour. It is of particular importance to note that sorghum flour used in this study was whole meal while wheat flour was refined. It is possible that the process of refining led to loss of minerals as the pericarp that contains most minerals was removed (Taylor & Anyango, 2011). The increase as a result of fortification may be due to the higher mineral content in RDP (Ssepuuya et al., 2019).

#### 3.2.1 Percentage contribution of mineral content of wheat–sorghum and wheat biscuits fortified with RDP per 100 g toward RDA for iron and zinc of children aged 0.5–10 years

Table 5 shows the contribution of wheat–sorghum and wheat biscuits fortified with defatted RDP per 100 g toward the Recommended Daily Allowance (RDA) of iron and zinc for children aged 0.5–10 years. The unfortified wheat biscuits did not meet half the RDA for iron and zinc for young children. Fortified wheat and wheat–sorghum biscuits were able to meet the iron requirements for 0.5–1‐year‐old children by 33% to 87% and 87% to 128%, respectively. Furthermore, they were able to meet zinc requirements by 167% to 573% for children 0.5–1 years of age, and 67% to 229% for 7–10‐year‐old children. Generally, the wheat–sorghum biscuits met iron and zinc requirements for school going children. The percentage contribution of the biscuits toward the iron and zinc decreased as the age of the children increased to 10 years. This can be attributed to increased nutrient requirements as the children get older (Rolfes et al., 2021). The contribution of iron and zinc above the RDA for 0.5–3‐year‐olds is within tolerable limits and not toxic, as iron and zinc toxicity is less likely to occur with dietary sources due to the body's ability to control their absorption (Food and Nutrition Board, 1989). Iron and zinc are micronutrients of public health significance among school going children in developing countries, as their deficiencies have detrimental effects on growth and development in this age group (Harika et al., 2017).

**TABLE 5:** Percentage contribution of mineral content of wheat–sorghum and wheat biscuits fortified with RDP per 100 g toward RDA for iron and zinc of children aged 0.5–10 years.

Mineral	Age group (years)	RDA^1^	% contribution of wheat–sorghum and wheat biscuits fortified with RDP per 100 g toward RDA									
			Wheat biscuits					Wheat–sorghum biscuits				
			0	5	15	25	40	0	5	15	25	40
Iron (mg/day)	0.5–1	11	33.2	37	63.1	66.5	86.7	73.5	86.7	100.8	116.4	127.5
	2–3	11.6	32.5	35.1	59.8	63.1	82.2	69.7	82.2	95.6	110.3	120.9
	4–6	12.6	28.9	32.3	55.1	58.1	75.7	64.2	75.7	88.0	101.6	111.3
	7–10	17.8	20.5	22.9	38.9	41.1	53.6	45.4	53.6	62.3	71.9	78.8
Zinc (mg/day)	0.5–1	2	139.5	167	266.5	383.5	444.5	221	291.5	383.5	429	573.5
	2–3	3	93	111.3	177.7	255.7	296.3	147.3	194.3	255.7	286	382.3
	4–6	5	55.8	66.8	106.6	153.4	177.8	88.4	116.6	153.4	171.6	229.4
	7–10	5	55.8	66.8	106.6	153.4	177.8	88.4	116.6	153.4	171.6	229.4

Abbreviation: RDA, recommended dietary allowance.

^1^ Rolfes et al. (2021).

### 3.3 Protein quality of flours and biscuits

Compositing wheat and wheat–sorghum with RDP increased the lysine content of biscuits made from both cereals (Table 6). *Ruspolia differens* powder had four times and six times lysine compared to wheat and sorghum flours, respectively. This can be attributed to the fact that the insect is rich in the essential amino acid at 54 to 69.8 mg/100 g protein (Ssepuuya et al., 2019). There was 11.4% to 148.6% increase and 29.6% to 222% increase in the lysine content of the biscuits compared to the 100% cereal biscuits, respectively. Similarly, Dewi et al. (2020) reported a 14.3% increase in lysine content of wheat biscuits fortified with wood grasshopper powder.

**TABLE 6:** The effect of compositing wheat and wheat–sorghum with *Ruspolia differens* powder on the lysine, reactive lysine content and in vitro protein digestibility (%) of flours and biscuits.

	Lysine (g/100 g biscuit or flour)	Lysine (g/100 g protein)^1^	Reactive lysine (g/100 g biscuit or flour)	Reactive lysine (g/100 g protein)	In vitro protein digestibility (%)
Flour					
Sorghum flour	0.26 ± 0.0^j^	2.28 ± 0.3^g^	0.13 ± 0.0^i^	0.14 ± 0.0^k^	49.14 ± 0.7^h^
Wheat flour	0.39 ± 0.0^h^	2.88 ± 0.0^g^	0.33 ± 0.0^h^	0.45 ± 0.0^j^	80.76 ± 2.3^b^
*Ruspolia differens* powder	1.59 ± 0.1^a^	3.20 ± 0.0^b^	0.68 ± 0.0^b^	3.39 ± 0.0^a^	65.17 ± 0.4^de^
Wheat:RDP biscuits					
100:00	0.35 ± 0.0^i^	2.71 ± 0.2^de^	0.34 ± 0.0^h^	0.44 ± 0.1^j^	54.94 ± 0.7^g^
95:5	0.39 ± 0.0^h^	2.65 ± 0.0^e^	0.38 ± 0.0^g^	0.56 ± 0.0^i^	65.50 ± 3.2^de^
85:15	0.50 ± 0.0^f^	2.76 ± 0.0^d^	0.43 ± 0.0^f^	0.79 ± 0.0^g^	72.77 ± 0.4^c^
75:25	0.79 ± 0.0^d^	3.12 ± 0.0^b^	0.52 ± 0.0^e^	1.31 ± 0.0^e^	79.00 ± 0.6^b^
60:40	0.87 ± 0.0^c^	3.45 ± 0.0^a^	0.61 ± 0.0^c^	1.72 ± 0.0^c^	83.89 ± 0.1^a^
Wheat–sorghum:RDP biscuits					
100:0	0.27 ± 0.0^j^	2.34 ± 0.2^g^	0.51 ± 0.0^e^	0.59 ± 0.0^i^	50.06 ± 3.9^h^
95:5	0.35 ± 0.0^i^	2.49 ± 0.0^f^	0.54 ± 0.0^d^	0.74 ± 0.0^h^	57.99 ± 1.4^f^
85:15	0.48 ± 0.0^g^	2.76 ± 0.0^de^	0.62 ± 0.0^c^	1.08 ± 0.0^f^	63.09 ± 0.5^e^
75:25	0.64 ± 0.0^e^	3.14 ± 0.0^b^	0.69 ± 0.0^b^	1.39 ± 0.0^d^	67.91 ± 0.7^d^
60:40	0.87 ± 0.0^c^	3.46 ± 0.0^a^	0.74 ± 0.0^a^	1.88 ± 0.0^b^	72.10 ± 1.9^c^

*Note*: Values are means ± standard deviation of three determinations. Values with the same letter superscript on the same column are not significantly different at (*p* < .05), as assessed by Fisher's least significant difference (LSD).

Abbreviation: RDP, *Ruspolia differens* powder.

^1^ Values calculated as follows: (100 g protein content × lysine content per 100 g)/(protein content per 100 g sample).

Compositing wheat and wheat–sorghum with RDP at levels of 5% to 40% increased the reactive lysine content in protein by 27.3% to 291% in wheat biscuits and 25.4% to 218.6% in wheat–sorghum biscuits compared to the 100% cereal biscuits (Table 6). Reactive lysine is the amount of lysine that can be absorbed in structural form for potential use in synthesis of proteins in the human body (Moughan & Rutherfurd, 2008). Studies on reactive lysine content of insect‐based foods are limited. However, studies using lysine‐rich legumes, such as soybean and cowpeas, show that increase in lysine results in an increase in the reactive lysine content of foods. Serrem et al. (2011) found that compositing sorghum and bread wheat with defatted soy flour at levels of 28.6% to 71.4% increased reactive lysine content in protein by 200% to 300% in sorghum biscuits and 4.5% to 9% in bread wheat biscuits compared to the 100% cereal biscuits. Fortification of sorghum *ugali* and *injera* with cowpea flour by Anyango et al. (2011) resulted in a 10%–75% increase in the reactive lysine content of the foods.

### 3.4 In vitro protein digestibility

Table 6 shows that compositing sorghum with RDP substantially increased in vitro protein digestibility (IVPD) of biscuits compared to the 100% cereal biscuits. The digestibility of the wheat–sorghum biscuits was generally lower than that of wheat biscuits. Fortification of the biscuits with RDP increased the IVPD by 14.6% to 42.5% in wheat–sorghum biscuits and 8.6% to 65.8% in wheat biscuits, respectively, relative to the 100% cereal biscuits. The increase in digestibility could be attributed to muscle proteins, myosin and actin, present in the RDP (Brogan et al., 2021). Insect proteins have been found to be highly digestible up to 90% (Manditsera et al., 2019; Mutungi et al., 2019). Similar findings have been reported by Bas and El (2022) who found a 200% increase in IVPD of wheat biscuits fortified with cricket powder compared to the control biscuits.

### 3.5 Amino acid composition

Table 7 shows the effect of compositing wheat and sorghum with *Ruspolia differens* powder on essential amino acid composition of biscuits and flour. Results show that fortification with RDP significantly improved the amino acid content of the biscuits. However, fortification with RDP had a marginal effect on improvement of the lysine score but it did not meet the reference pattern for children aged 3–10 years (Table 8). This can be attributed to the loss of lysine due to processing and also baking and also to the in vitro digestibility values for RDP and wheat. For instance, the lysine content of the processed RDP was 31.98 mg/g protein, which is almost half of what has been reported in unprocessed *Ruspolia differens* sample of 54–69.8 mg/g protein (Ssepuuya et al., 2019). This could be a result of Maillard glycation, protein denaturation, and loss of lysine in the formation of Maillard reaction products (Ochieng et al., 2022), as explained earlier. The marginal improvement in the in vitro digestibility and the lysine score improved the Protein Digestibility Corrected Amino Acid Score (PDCAAS). Fortification with RDP improved the PDCAAS by 2% to 33% and 6% to 47% in wheat and wheat–sorghum biscuits, respectively.

**TABLE 7:** The effect of compositing wheat and wheat–sorghum with *Ruspolia differens* powder on essential amino acid composition of biscuits and flour (mg/g protein).

	Histidine	Isoleucine	Leucine	Lysine	Met+Cye^1^	Phe+Tyr^2^	Threonine	Tryptophan	Valine
Flour									
Sorghum flour	23.36^3^ ± 0.3^i^ 1.5^4^	34.18 ± 1.6^i^ 1.1	98.36 ± 1.0^h^ 1.6	22.77 ± 2.2^g^ 0.5	31.14 ± 1.6^h^ 1.4	71.34 ± 0.0^g^ 1.7	35.29 ± 1.5^h^ 1.4	14.40 ± 0.1^i^ 2.2	49.85 ± 0.0^i^ 1.2
Wheat flour	20.31 ± 0.1^k^ 1.3	40.80 ± 0.1^e^ 1.4	117.68 ± 0.6^e^ 1.9	28.76 ± 0.4^c^ 0.6	32.00 ± 0.6^gh^ 1.4	73.36 ± 0.1^e^ 1.8	40.62 ± 0.0^c^ 1.6	12.03 ± 0.0^k^ 1.8	53.42 ± 0.0^f^ 1.3
*Ruspolia differens* powder	19.02 ± 0.7^L^ 1.2	44.30 ± 0.9^c^ 1.5	88.62 ± 1.9^i^ 1.5	31.98 ± 1.0^b^ 0.7	18.91 ± 0.4^i^ 0.8	84.37 ± 0.4^a^ 2.1	33.23 ± 1.1^i^ 1.3	9.77 ± 0.2l 1.5	59.85 ± 1.1^c^ 1.5
Biscuits Wheat:RDP biscuits									
100:00	22.31 ± 0.1^j^ 1.4	37.8 ± 0.1^g^ 1.3	115.01 ± 0.6^f^ 1.9	25.76 ± 0.4^ef^ 0.5	33.37 ± 0.5^f^ 1.4	71.36 ± 0.1^g^ 1.7	37.62 ± 0.0^fg^ 1.5	14.03 ± 0.0^j^ 2.1	51.42 ± 0.0^h^ 1.2
95:5	24.81 ± 0.2^h^ 1.6	39.36 ± 0.3^fg^ 1.3	117.85 ± 0.3^e^ 1.9	26.49 ± 0.4^de^ 0.6	34.67 ± 0.1^e^ 1.5	71.97 ± 0.0^f^ 1.8	38.47 ± 0.1^ef^ 1.5	15.12 ± 0.2^h^ 2.2	52.62 ± 0.2^g^ 1.3
85:15	26.36 ± 0.3^f^ 1.6	41.37 ± 0.0^d^ 1.4	120.19 ± 0.1^d^ 1.9	27.62 ± 0.5^cd^ 0.6	36.14 ± 0.0^d^ 1.6	73.59 ± 0.1^e^ 1.8	39.82 ± 0.0^cd^ 1.6	16.26 ± 0.1^f^ 2.5	55.93 ± 0.0^e^ 1.4
75:25	29.35 ± 0.1^d^ 1.8	43.24 ± 0.1^b^ 1.4	122.71 ± 0.0^c^ 2.0	31.24 ± 0.0^b^ 0.7	37.34 ± 0.0^c^ 1.6	75.02 ± 0.0^d^ 1.8	42.52 ± 0.0^b^ 1.7	18.45 ± 0.0^d^ 2.8	56.86 ± 0.1^d^ 1.4
60:40	33.63 ± 0.0^b^ 2.1	46.57 ± 0.0^b^ 1.6	127.29 ± 0.0^a^ 2.1	34.48 ± 0.0^a^ 0.7	39.39 ± 0.1^a^ 1.7	77.02 ± 0.2^c^ 1.9	43.29 ± 0.1^b^ 1.7	19.27 ± 0.0^c^ 2.9	60.26 ± 0.0^c^ 1.5
Wheat–sorghum:RDP biscuits									
100:0	23.36 ± 0.3^i^ 1.5	35.84 ± 0.1^h^ 1.2	110.36 ± 0.0^g^ 1.8	23.46 ± 0.1^g^ 0.5	32.80 ± 0.0^fg^ 1.4	71.34 ± 0.0^g^ 1.7	36.95 ± 0.0^g^ 1.5	14.40 ± 0.1^i^ 2.1	49.85 ± 0.0^i^ 1.2
95:5	25.28 ± 0.1^g^ 1.6	38.58 ± 0.1^fg^ 1.3	115.44 ± 0.9^f^ 1.9	24.91 ± 0.0^f^ 0.5	34.79 ± 0.1^e^ 1.5	73.48 ± 0.0^e^ 1.8	37.64 ± 0.0^fg^ 1.5	15.86 ± 0.0^g^ 2.4	51.39 ± 0.0^h^ 1.2
85:15	27.64 ± 0.0^e^ 1.7	42.59 ± 0.0^d^ 1.4	118.41 ± 0.0^e^ 1.9	27.62 ± 0.6^d^ 0.6	37.60 ± 0.0^c^ 1.6	74.89 ± 0.0^d^ 1.8	39.28 ± 0.0^de^ 1.6	17.91 ± 0.0^e^ 2.7	56.86 ± 0.0^d^ 1.4
75:25	30.62 ± 0.0^c^ 1.9	46.81 ± 0.0^b^ 1.6	121.88 ± 0.7^c^ 2.0	31.44 ± 0.0^b^ 0.7	38.52 ± 0.4^b^ 1.7	76.95 ± 0.0^c^ 1.9	42.92 ± 0.1^b^ 1.7	22.98 ± 0.0^b^ 3.5	61.85 ± 0.2^b^ 1.5
60:40	36.03 ± 0.3^a^ 2.2	50.65 ± 0.3^a^ 1.7	125.95 ± 0.5^b^ 2.0	34.57 ± 0.1^a^ 0.7	39.96 ± 0.0^a^ 1.7	80.32 ± 0.0^b^ 2.0	46.36 ± 0.1^a^ 1.9	25.19 ± 0.0^a^ 3.8	64.62 ± 0.7^a^ 1.6
Reference pattern^5^	**16**	**30**	**61**	**48**	**23**	**41**	**25**	**6.6**	**40**

*Note*: Values are means ± standard deviation of three determinations. Values with the same letter superscript on the same column are not significantly different at (*p* < .05), as assessed by Fisher's least significant difference (LSD).

Abbreviation: RDP, *Ruspolia differens* powder.

^1^ Methionine + cysteine.

^2^ Phenylalanine + tyrosine.

^3^ Amino acid concentration (mg/g protein).

^4^ Amino acid score = mg amino acid in 1 g protein of sample/ mg amino acid in requirement pattern (WHO/FAO/UNU Expert Consultation, 2007) for children 3–10 years.

^5^ Pattern for amino acid requirements for children aged 3–10 years (WHO/FAO/UNU Expert Consultation, 2007) are shown in bold.

**TABLE 8:** Indispensable amino acid composition (mg/g protein) of wheat and wheat–sorghum biscuits fortified with *Ruspolia differens* compared with the pattern for amino acid requirements (mg/g crude protein) for children aged 3–10 years.

Amino acids	Fortification levels with *Ruspolia differens* powder										Reference pattern^1^
	Wheat biscuits					Wheat–sorghum biscuits					
	0	5	15	25	40	0	5	15	25	40	
Histidine	22.31	24.81	39.82	29.35	33.63	23.36	25.28	27.64	30.62	36.03	16
Isoleucine	37.8	39.36	41.37	43.24	46.57	35.84	38.58	42.59	46.81	50.65	30
Leucine	115.01	117.85	120.19	122.71	127.29	110.36	115.44	118.41	121.88	125.95	61
Lysine	25.76	26.49	27.62	31.24	34.48	23.46	24.91	27.62	31.44	34.57	48
Met+Cysteine^2^	33.37	34.67	36.14	37.34	39.39	32.8	34.79	37.6	38.52	39.96	23
Phe+Tyr^3^	71.36	71.97	73.59	75.02	77.02	71.34	73.48	74.89	76.95	80.32	41
Threonine	37.62	38.47	39.82	42.52	43.29	36.95	37.64	39.28	42.92	46.36	25
Tryptophan	14.03	15.12	16.26	18.45	19.27	14.4	15.86	17.91	22.98	25.19	6.6
Valine	51.42	52.62	55.93	56.86	60.26	49.85	51.39	56.86	61.85	64.62	40
Protein (%)	12.97	14.9	18.27	25.13	28.01	11.6	13.91	17.44	20.33	25.29	
Total	408.68	421.36	450.74	456.73	481.2	398.36	417.37	442.8	473.97	503.65	
IVPD (%)	55	66	73	79	84	50	58	63	67	72	
Limiting AA	Lysine	Lysine	Lysine	Lysine	Lysine	Lysine	Lysine	Lysine	Lysine	Lysine	
Lysine score	0.54	0.55	0.58	0.65	0.72	0.49	0.52	0.58	0.66	0.72	
PDCAAS^4^	0.27	0.34	0.42	0.52	0.6	0.27	0.32	0.36	0.44	0.52	

Abbreviations: AA, amino acid; IVPD, in vitro protein digestibility; PDCAAS, Protein Digestibility Corrected Amino Acid Score.

^1^ Amino acid reference pattern for children aged 3–10 years (WHO, 2007).

^2^ Met+ Cys, Met+Cysteine: methionine + cysteine (sulfur AA).

^3^ Phe + Tyr: phenylalanine + tyrosine.

^4^ PDCAAS based on IVPD (rounded off to the nearest whole figure).

Studies on the use of edible insect powder to improve the PDCAAS of cereal‐based foods are lacking. However, studies using legumes indicate that fortification significantly improves the protein quality of the foods. Anyango et al. (2011) reported two to threefold improvement in the PDCAAS of sorghum‐based foods fortified with cowpea flour. Similarly, Serrem et al. (2011) established that fortifying sorghum and bread wheat flours with soy flour at 28.6% to 71.4% levels increased PDCAAS by 7 to 10 times and 2 to 2.5 times, respectively, in biscuits. In these studies, the improved PDCAAS was a result of improvement in lysine and protein digestibility. Similarly, Agengo et al. (2020) established that fortification of sorghum buns using snail meat powder improved the PDCAAS by 20% to 73%.

### 3.6 Consumer acceptability

Table 9 shows consumer perceptions of sensory attributes of RDP‐fortified wheat–sorghum and wheat biscuits. The appearance, smell, flavor, and texture of all, except the 40% RDP biscuits, were liked by consumers. The appearance of the pure wheat biscuit scored the highest, while the 40% RDP scored the lowest. All other fortified wheat and wheat–sorghum biscuits were liked equally, with only a difference of 8% to 13% from the reference wheat biscuit. The low score for the 40% RDP biscuit may be attributed to the consumers' unfamiliarity with the increased dark color of biscuits as the level of RDP increased. Studies have shown that increasing the level of insect incorporation into food products negatively affects acceptability (Bawa et al., 2020; González et al., 2019). The flavor of the 40% RDP biscuit was also the lowest. It is likely that undesirable volatile aroma compounds resulting from lipid oxidation were perceived by the consumers. Such organic compounds commonly found in *Ruspolia differens* oil are carboxylic acids (2‐methylbutanoic acid and 3‐methylbutanoic acid), aldehydes (heptanal, octanal, nonanal, and furfural), and alcohols (pentanols) (Cheseto et al., 2020; Ssepuuya et al., 2020). A similar study, by Pozo‐Bayón et al. (2006), concluded that 2‐ and 3‐methylbutanoic acids and octanal were responsible for sweaty and soapy odors, respectively, in baked goods.

**TABLE 9:** Effect of fortification with *Ruspolia differens* powder on consumer perception of wheat–sorghum and wheat biscuit sensory attributes.

Attributes	*Ruspolia differens* powder (RDP) level (%)									
	Wheat–sorghum biscuits					Wheat biscuits				
	0	5	15	25	40	0	5	15	25	40
Appearance	6.90 ± 1.9^bc^	6.97 ± 1.9^b^	6.64 ± 1.9^bc^	6.65 ± 1.9^bc^	6.40 ± 2.2^cd^	7.59 ± 1.8^a^	6.98 ± 2.2^b^	6.91 ± 1.8^bc^	6.68 ± 1.8^bc^	6.11 ± 2.2^d^
Smell	6.72 ± 1.6^b^	6.74 ± 1.8^b^	6.13 ± 2.1^cd^	5.89 ± 2.3^d^	5.22 ± 2.2^e^	7.54 ± 1.6^a^	6.95 ± 1.9^b^	6.53 ± 2.1^bc^	6.15 ± 2.2^cd^	4.84 ± 2.5^e^
Flavor	6.50 ± 1.9^bcd^	6.84 ± 1.7^ab^	5.87 ± 2.3^e^	5.77 ± 2.3^de^	5.26 ± 2.3^fg^	7.31 ± 1.8^a^	6.22 ± 2.4^cde^	6.68 ± 2.1^bc^	5.98 ± 2.1^de^	4.85 ± 2.5^g^
Texture	6.06 ± 2.1^cd^	6.31 ± 1.9^bcd^	5.98 ± 2.2^d^	6.02 ± 2.2^d^	5.78 ± 2.2^d^	7.27 ± 1.7^a^	6.75 ± 1.8^ab^	6.58 ± 1.9^bc^	6.32 ± 2.0^bcd^	5.79 ± 2.3^d^
Aftertaste	6.13 ± 2.3^bc^	6.21 ± 2.0^b^	5.57 ± 2.5^cde^	5.47 ± 2.5^de^	4.96 ± 2.3^ef^	7.24 ± 1.9^a^	6.08 ± 2.4^bcd^	6.43 ± 2.3^b^	5.98 ± 2.4^bcd^	4.66 ± 2.6^f^

*Note*: Values are means ± SD. Values followed by different letter superscripts in a row are significantly different at *p* < .05, as assessed by Fisher's least significant test. Sensory attributes score; 9 = Like extremely, 8 = Like very much, 7 = Like moderately, 6 = Like slightly, 5 = Neither like nor dislike, 4 = Dislike slightly, 3 = Dislike moderately, 2 = Dislike very much, 1 = Dislike extremely; *n* = 110.

Abbreviation: RDP, *Ruspolia differens* powder.

The texture of the wheat biscuits was scored higher than that of wheat–sorghum biscuits. Also, the biscuits with a high RDP content had less desirable textures. It is possible that liking of sorghum‐based biscuits, and those fortified with higher RDP levels, decreased due to the rough, grainy, and coarse textures perceived in biscuits by consumers. The roughness was probably caused by the pericarp of the sorghum grain (Banerjee et al., 2014) and the chitin of the grasshopper exoskeleton (Rumpold & Schlüter, 2013). Results consistent with reduced score for textural attributes of insect‐based foods have been reported by Ochieng et al. (2023) while working with cookies enriched with *Ruspolia differens* powder.

## 4 CONCLUSIONS

Compositing wheat and sorghum–wheat biscuits with RDP improves the nutritional qualities of the biscuits, where they met half of the protein and the mineral requirements for children aged 0.5–10 years. The fortified biscuits were liked by the consumers. The wheat and wheat–sorghum composite biscuits fortified with RDP have considerable potential as protein‐rich supplementary foods to alleviate protein and energy malnutrition in children.

## AUTHOR CONTRIBUTIONS

**Amos Kipkemoi Ronoh:** Conceptualization (equal); data curation (equal); formal analysis (equal); funding acquisition (equal); investigation (equal); methodology (equal); resources (equal); software (equal); writing – original draft (equal); writing – review and editing (equal). **Charlotte Atsango Serrem:** Conceptualization (equal); data curation (equal); formal analysis (equal); funding acquisition (equal); investigation (equal); methodology (equal); resources (equal); software (equal); supervision (equal); validation (equal); visualization (equal); writing – original draft (equal); writing – review and editing (equal). **Susan Balaba Tumwebaze:** Conceptualization (equal); data curation (equal); formal analysis (equal); funding acquisition (equal); investigation (equal); methodology (equal); resources (equal); software (equal); supervision (equal); validation (equal); visualization (equal); writing – original draft (equal); writing – review and editing (equal). **Gertrude Mercy Were:** Conceptualization (equal); data curation (equal); formal analysis (equal); funding acquisition (equal); investigation (equal); methodology (equal); resources (equal); software (equal); supervision (equal); validation (equal); visualization (equal); writing – original draft (equal); writing – review and editing (equal).

## CONFLICT OF INTEREST STATEMENT

The authors declare that they have no known competing interests.

## Data Availability

The data that support the findings of this study are available from the corresponding author upon reasonable request.
